# Identification and Characterization of Buffalo *7SK* and *U6* pol III Promoters and Application for Expression of Short Hairpin RNAs

**DOI:** 10.3390/ijms15022596

**Published:** 2014-02-14

**Authors:** Xiaoxi Zhang, Qingyou Liu, Chan Luo, Yanfei Deng, Kuiqing Cui, Deshun Shi

**Affiliations:** 1 State Key Laboratory for Conservation and Utilization of Subtropical Agro-Bioresources, Guangxi University, 75 Xiuling Road, Nanning 530005, China; E-Mails: xiaoxizhang527@gmail.com (X.Z.); qyliu2002@gmail.com (Q.L.); luochan2000@163.com (C.L.); yanfei-dun@163.com (Y.D.); kqcui@126.com (K.C.); 2 Faculty of Basic Medicine, Guilin Medical University, Huan Cheng North 2nd Road 109, Guilin 541004, China

**Keywords:** buffalo, *7SK* and *U6* promoter, shRNA

## Abstract

RNA polymerase III (pol III) type 3 promoters, such as *7SK* and *U6*, are routinely used to induce short hairpin RNAs (shRNAs) to knockdown gene expression by RNA interference (RNAi). To extend the application of RNAi to studies of buffalo, an shRNAs expressing system using the buffalo pol III promoters was developed. Buffalo *7SK* promoter (bu*7SK*) and *U6* promoter (bu*U6*) sequences upstream of the full-length *7SK* and *U6* small nuclear RNA sequence in the buffalo genome were identified and characterized, respectively. To determine the functionality of these promoters in constructs driving shRNA expression, anti-EGFP shRNAs (shEGFP) cassettes under the direction of bu*7SK* and bu*U6* were constructed. We further compared the EGFP knockdown efficiency of constructs using bu*7SK* and bu*U6* with that of promoters of human and bovine origins in BFF cells and mouse PT67 cells by flow cytometry and quantitative real-time PCR assays. We found that the bu*7SK* and bu*U6* promoters induced the greatest level of suppression in homologous and heterologous cells relative to promoters derived from other species. Taken together, functional bu*7SK* and bu*U6* promoters were identified and characterized, thus laying the groundwork for future development of RNAi therapeutics and gene modification in buffalo species.

## 1. Introduction

RNA interference (RNAi), a natural and conserved sequence-specific gene-silencing mechanism [1], has been used to analyze gene function in plants, invertebrates, and more recently mammalian cells, with the use of 19–29 nt duplexes known as small-interfering RNAs (siRNAs) [2]. Recently, the development of DNA-based vectors for expression of short hairpin RNAs (shRNAs), which are processed within the cell to produce active siRNA molecules, has progressed rapidly [3,4]. The shRNAs are transcribed from these vectors as 19–29 nt inverted repeat sequences separated by a 4–10 nt loop sequence that fold spontaneously to form hairpin structures, which are subsequently cleaved by Dicer into siRNAs [3,5]. RNA polymerase III (pol III) type 3 promoters are most commonly chosen for expressing the shRNAs [5], as these promoters naturally direct the synthesis of small, highly abundant non-coding RNA transcripts such as *7SK* and *U6* [6,7]. Unlike type 1 and 2 promoters, pol III type 3 promoters are located entirely upstream of transcription start sites (+1), with a TATA box beginning at around −30 bp (relative to +1), a proximal sequence element (PSE) centered around −60 bp and a distal sequence element (DSE) beginning around −240 bp [6]. In the human *U6* (h*U6*) and *7SK* (h*7SK*) promoters, the DSE is comprised of at least one Octamer (OCT) motif [8,9] and an SphI Post*-*octamer Homology (SPH) domain [10–12]. The DSE of the human *7SK* (h*7SK*) also contains an additional CACCC box enhancer located between the OCT and SPH elements [13]. The human *U6* has characteristic promoter elements (for example, OCT-1, SPH, PSE and TATA box, *etc.*) known as enhancer and core regions [14–16], and is frequently used in shRNA expression vectors [17].

Water buffalo (*Bubalus bubalis*) are important domestic animals distributed in the tropical and subtropical regions. They provide more than 5% of the world’s milk supply and 20% to 30% of the farm power in Southeast Asia [18,19]. Recently, the majority of buffalo labor has been replaced by machines in Southeast Asia, and the governments have mated swamp buffalo with river buffalo, developed a buffalo clone [20] and employed genetic modification techniques to change the properties of buffalo to yield more milk and meat. In the current study, the buffalo *7SK* and *U6* promoters (bu*7SK* and bu*U6*) were identified and characterized. Further, they were shown to be suitable for driving shRNAs in homologous and heterologous cells lines, laying the groundwork for future development of RNAi technology and gene modification in buffalo species.

## 2. Results

### 2.1. Identification and Isolation of Buffalo *7SK* and *U6* Promoters

**Figure 1.**
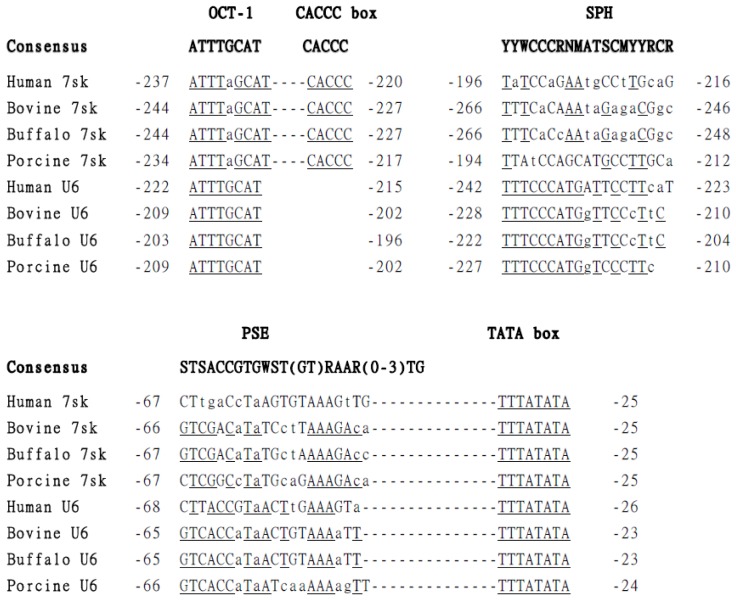
Sequence alignment of promoter elements in the buffalo, bovine, human and porcine *7SK* and *U6* promoters. The distal promoter regions containing the SphI Post-octamer Homology (SPH), Octamer (OCT)-1 and CACCC box sequences and proximal promoter regions containing the proximal sequence element (PSE) and TATA sequence elements are shown for each promoter. Matches to the consensus sequence, delineated at the top of the SPH [12,13], OCT-1 [21] and PSE [22] sequences, are shown in upper case. Nucleotide positions indicate the location (5′→3′) of each element in the promoter relative to the transcription start site (+1). Each dash mark between the OCT-1 and CACCC box, PSE and TATA box represents one nucleotide. Nucleotide abbreviations in consensus sequences are according to the International Union of Biochemistry convention for GenBank (http://www.ncbi.nlm.nih.gov/).

To locate the *7SK* promoter (bu*7SK*) in the buffalo genome, we employed a bioinformatics approach, scanning the bovine genome with the 331 nt human *7SK* snRNA sequence (GenBank accession number NR001445) as a query. A bovine sequence (the region from 10950489 to 10950820 in NW_003104551.1) showing similarity (99%) to human *7SK* snRNA was selected. Using the predicted bovine sequence as reference, the bu*7SK* included fragment was amplified from the buffalo genome. Sequenced and analyzed the amplified fragment, a 432 bp at 5′ flanking regions of bu*7SK* snRNA sequence was identified as novel bu*7SK* promoter (GenBank accession number JN417658) by the presence of pol III promoter elements, including a TATA box at bp −32 to −25, a PSE at bp −67 to −47, a SPH domain at bp −266 to −248 and an OCT motif at bp −244 to −227. The same method was used to clone and identify the 400 bp buffalo *U6* promoter (bu*U6*) (GenBank accession number JN417659), which contains the typical elements, including a TATA box at bp −31 to −24, a PSE at bp −66 to −46, a SPH at bp −222 to −204 and an OCT motif at bp −203 to −196. The TATA box, PSE, OCT and SPH elements displayed considerable homology to published consensus sequences through alignment of the bu*7SK* and bu*U6* sequences with the respective promoters of other species (Figure 1).

### 2.2. Construction and Validation of shRNA Expression Vectors

**Figure 2.**
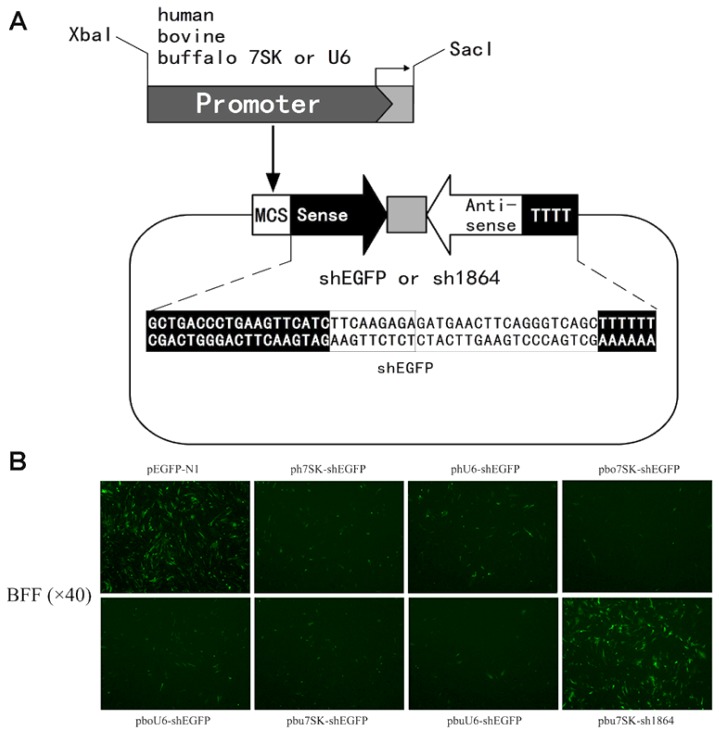
Construction and transfection of dual EGFP/shRNA expression vectors. (**A**) Buffalo, bovine and human *7SK*/*U6* promoter fragments were amplified with restriction enzymes sites *Xba*I/*Sac*I introduced by the primers, and ligated into the 5′ MCS (multi-cloning site) of the pMCS-shEGFP and pMCS-sh1864 vectors. The core fragments in the vectors, featuring the MCS and sense, loop, antisense and pol III terminator (TTTTTT), were chemically synthesized and ligated with framework plasmid pMD18-T; (**B**) Fluorescence microscopy images of buffalo fetal fibroblasts (BFF) cells transfected with the reporter vector (pEGFP-N1) only, or co-transfected with the reporter and various shEGFP expression plasmids as indicated in each image.

In order to validate their function, the putative bu*7SK* and bu*U6* promoter sequences were used to construct shRNA expression vectors, termed pbu*7SK*-shEGFP and pbu*U6*-shEGFP, which were designed to drive the expression of shRNAs targeting EGFP (shEGFP) (Figure 2A). Another vector, pbu*7SK*-sh1864, was constructed to drive the expression of a control scrambled shRNA. In addition, EGFP-targeting shRNA expression vectors ph*7SK*-shEGFP, ph*U6*-shEGFP, pbo*7SK*-shEGFP and pbo*U6*-shEGFP were constructed with the cloned *7SK* and *U6* promoters of human (h), and bovine (bo), respectively (Figure 2A).

### 2.3. The bu*7SK* and bu*U6* Promoters Direct shRNA-Mediated Knockdown

To determine RNAi-mediated knockdown efficiency of the above constructs, we transfected pbu*7SK*-shEGFP, pbu*U6*-shEGFP, ph*7SK*-shEGFP, ph*U6*-shEGFP, pbo*7SK*-shEGFP and pbo*U6*-shEGFP into Buffalo fetal fibroblasts (BFF) cells along with an EGFP expression vector (pEGFP-N1). According to Figure 2B, EGFP silencing was clearly observed by fluorescence microscopy in cells transfected with shEGFP expression vectors, with the exception of the negative control group. The EGFP knockdowns induced by the bu*7SK*-shEGFP and bu*U6*-shEGFP constructs were comparable to those of other constructs, which indicates shRNA expressing function of cloned buffalo promoters (Figure 2B).

**Figure 3.**
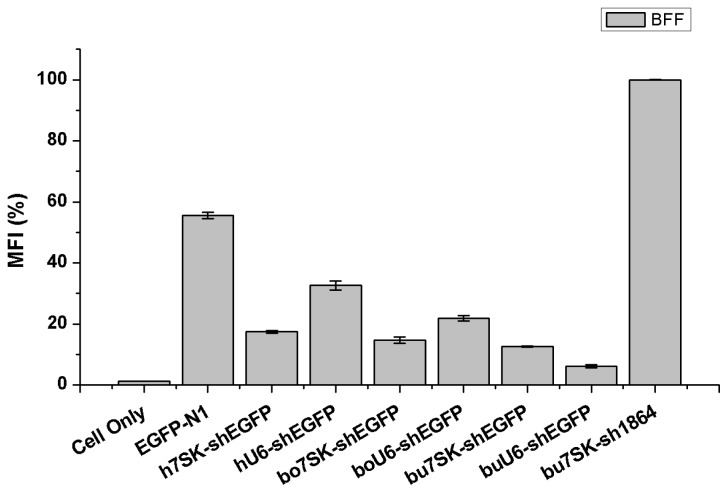
Flow cytometry analysis of EGFP knockdown in BFF cells. The mean fluorescence intensity (MFI) of BFF cells 72 h post-transfection was determined by flow cytometry. EGFP knockdown is presented as percent MFI, normalized to the average MFI of the negative control cell group with pEGFP-N1 co-transfected with pbu*7SK*-sh1864 (100%). Error bars represent the standard error of the mean (SEM) calculated from three independent experiments.

To further compare the EGFP silencing efficiency among the shEGFP expression constructs, flow cytometry was performed and the mean fluorescence intensity (MFI) from triplicate co-transfections for each condition was recorded (Figure 3). The MFI observed with co-transfection of the reporter pEGFP-N1 and the negative control pbu*7SK*-sh1864 construct was considered to be 100% [23], and we considered any reduction in EGFP fluorescence intensity to reflect RNAi-mediated EGFP knockdown due to the activity of the shRNA expression constructs. The EGFP MFI of the pbu*7SK*-shEGFP and pbu*U6*-shEGFP co-transfection groups were reduced about 87.45% (±0.16%) and 93.82% (±0.52%), respectively, compared with the pEGFP-N1 + pbu*7SK*-sh1864 co-transfection group. This reduction was significantly greater than that induced by ph*7SK*-shEGFP (82.55% ± 0.46%), ph*U6*-shEGFP (67.34% ± 1.48%) and pbo*U6*-shEGFP (78.14% ± 0.90%, *p* < 0.05) (Figure 3). These results indicate that the shEGFP molecules expressed by the bu*7SK* and bu*U6* promoters could direct efficient knockdown (>85%) of EGFP in BFF cells to an extent even greater than that of the commonly used h*7SK* and h*U6* promoters.

### 2.4. Comparison of Knockdown Efficiency by qRT-PCR

**Figure 4.**
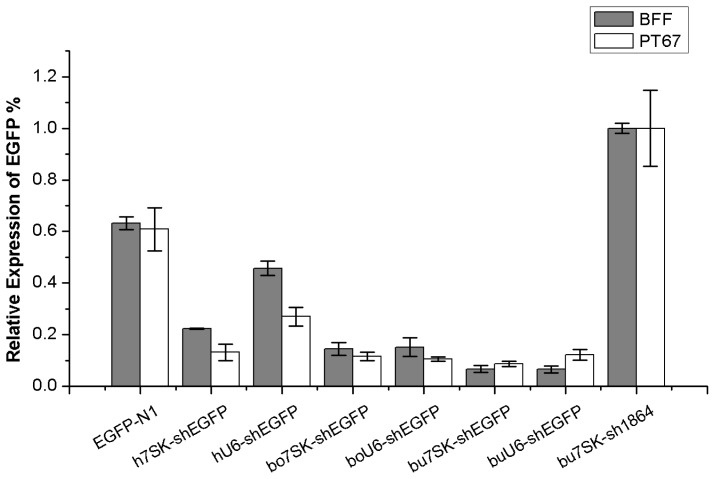
Detection of EGFP knockdown efficiency by qRT-PCR. The expression of EGFP in BFF cells is labeled as grey and in PT67 cells as white. EGFP knockdown efficiency in different transfection groups is presented as a percent relative expression detected by qRT-PCR, normalized to the average relative expression of the negative control pbu*7SK*-sh1864 (100%). Error bars represent the standard error of the mean (SEM) calculated from four independent experiments.

The silencing efficiency of EGFP expression in different groups was also assayed by quantitative real-time PCR (qRT-PCR). The expression of EGFP was normalized to the relative expression level of the pbu*7SK*-sh1864-tranfected negative control cells (considered to be 100%). The plotting of relative EGFP expression levels and statistical analyses (Figure 4) indicate that the EGFP knockdown induced by pbu*7SK*-shEGFP construct (93.29% ± 1.30%) was comparable to that induced by pbu*U6*-shEGFP (93.45% ± 1.25%), but significantly greater than that conferred by ph*7SK*-shEGFP (77.66% ± 0.20%) and ph*U6*-shEGFP (54.28% ± 2.73%) (*p* < 0.001). These results indicated that both of the buffalo promoter-shEGFP constructs induced more efficient RNAi-mediated knockdown of EGFP than other existing shEGFP expression constructs in BFF cells. In PT67 cells, pbu*7SK*-shEGFP co-transfected cells showed the greatest reduction in EGFP expression (91.29% ± 1.03%), which was significantly higher than that of ph*U6*-shEGFP (73.00% ± 3.63%, *p* < 0.05), and not significantly different than that of the ph*7SK*-shEGFP (86.84% ± 3.10%), pbo*7SK*-shEGFP (88.38% ± 1.59%), pbo*U6*-shEGFP (89.50% ± 0.81%) and pbu*U6*-shEGFP (87.73% ± 2.03%) co-transfected cells (*p* > 0.05, Figure 4).

## 3. Discussion

Although several recently characterized chicken, bovine and porcine *7SK* and *U6* promoters have been used to develop effective shRNA expression systems, whether shRNA promoters in buffalo could be employed for similar use was a lingering question. Buffalo are an integral component of traditional Asian agriculture through their contributions to milk, meat and draft power [24], and thus play a pivotal role in agriculture in several Asian countries [25], especially in South China. To date, buffalo and bovine-related RNAi research has focused on silencing viruses, such as foot-and-mouth disease virus (FMDV) and rinderpest virus (RPV), and the knockdown of genes that influence reproductive performance such as inhibin-A [26–29]. To the best of our knowledge, we are the first to identify and characterize the buffalo RNA polymerase III promoters and demonstrate their functionality for shRNA expression.

In the human, bovine, chicken and mouse genomes, functional *7SK* promoters have been identified upstream of *7SK* snRNA coding sequences [30–34]. It was likely that a functional promoter could be located upstream of the buffalo *7SK* snRNA sequences. Following systematic analysis of the bovine *7SK* genomic sequence, a 432 bp bu*7SK* promoter fragment was cloned from buffalo genomic DNA. The multiple alignment results showed that bu*7SK* had typical pol III promoter motifs, including TATA, PSE, OCT-1 and SPH elements, which show positional and sequence similarities to those of the h*7SK*, b*7SK* and p*7SK* promoters [8,31,35]. Using a similar strategy, a 400 bp bu*U6* promoter was identified in the buffalo genome, and is believed to be the buffalo orthologous of the bovine *U6* snRNA promoter. We found that the TATA and OCT-1 motifs were highly conserved in human, bovine, buffalo and porcine *7SK* and *U6* promoters, while there were extensive variations in the PSE and SPH motifs within different species, which is consistent with a report by Cummins *et al.* [35].

To determine the activity of buffalo pol III promoters bu*7SK* and bu*U6*, shRNA expression vectors targeting EGFP were constructed (pbu*7SK*-shEGFP, pbu*U6*-shEGFP). To compare the silencing efficiency and specificity of these vectors, the *7SK* and *U6* promoters of human and bovine were also cloned to construct corresponding shRNA expression vectors (ph*7SK*-shEGFP, ph*U6*-shEGFP, pbo*7SK*-shEGFP and pbo*U6*-shEGFP). These constructs were co-transfected with pEGFP-N1 (an EGFP expression vector) into homologous (BFF) and heterologous (PT67) cell lines, and the gene silencing efficiency of the different promoter constructs was compared. The expression of EGFP was clearly reduced in the groups of cells co-transfected with the reporter plasmid and the buffalo promoter constructs, which indicate that bu*7SK* and bu*U6* are functional promoters in this RNAi system. We are excited to report that buffalo pol III promoters demonstrate significant shRNA expression abilities in both BFF cells and PT67 cells. This difference in efficiency is similar to a study by Lambeth *et al.*, which demonstrated that the bovine *7SK* promoter was far more effective than a mouse *U6* promoter-based shRNA expression vector in bovine cells [31]. Previous studies [31,32,34,35] have explored the issue of whether promoters perform more efficiently in cell lines that are of the same species as the origin of the promoter. In this study, greater expression was achieved when utilizing buffalo promoters in buffalo cells than in mouse cells, indicating pol III promoters drives efficient shRNA transcription with species specificity. Interestingly, the buffalo promoters showed greater silencing efficiency than human promoters in mouse cells, suggesting that the bu*7SK* and bu*U6* promoters can also be used in investigations of other species employing shRNA-mediated knockdown.

These observations correspond with related studies finding that optimal efficiency is not dependent on the presence of an SPH region [36], but rather can be related to the relative location of the OCT-1 and SPH elements [12,16], as well as the downstream spacing and sequence of OCT-1 motif [35]. The features distinguishing the bu*7SK* from the bo*7SK* PSE are the presence of a G > C substitution at bp −57, an A > T substitution at bp −54 and a C > A substitution at bp −47 of the bu*7SK* PSE motif. For the *U6* promoter, the sequence of elements OCT-1, SPH, PSE and TATA of buffalo and bovine *U6* promoter are exactly the same; however, relative to the buffalo *U6* promoter, bovine elements are located an additional 6 bp upstream of the start site. The OCT-1 and SPH element of buffalo, bovine and porcine *U6* promoters are of a similar distance from the start site, but the human OCT-1 element was found to be 13~17 bp farther from +1 site than the other three *U6* promoters. This may be one of the reasons that human *U6*-mediated expression was not as strong as the other promoters.

## 4. Experimental Section

### 4.1. Isolation of the bu*7SK* and bu*U6* Promoter from Buffalo Genomic DNA

The bu*7SK* and bu*U6* promoter sequences were amplified from buffalo genomic DNA extracted from the blood of Chinese swamp buffalo, using the bu*7SK*-F/R (5′-GAG ACA GAC CTG GCT CCA C-3′ and 5′-CAC ATC CGA GAC ACT CTG C-3′) and bu*U6*-F/R (5′-GAG CAT TCA GTC CGG CAG-3′ and 5′-GCA CGG TAA ACA TGG CTT C-3′) primer pairs. Gradient PCR was conducted using 9.5 ng of genomic DNA, 100 ng of each primer (forward and reverse), 2 mM MgCl_2_ (Sigma-Aldrich, Milwaukee, WI, USA), 250 μM dNTPs (Takara, Dalian, China), 1× PCR buffer (Takara, Dalian, China) and 1 unit of Taq polymerase (Takara, Dalian, China). Thermocycling was performed in a Master cycler EP Gradient S thermocycler (Eppendorf AG, Hamburg, Germany) under the following conditions: 94 °C for 3 min; 30 cycles of 94 °C for 30 s, 58 °C for 30 s and 72 °C for 30 s; and a final extension at 72 °C for 2 min.

The promoters of bu*7SK* (432 bp) and bu*U6* (400 bp) were amplified by PCR, purified using the QIAquick Gel Extraction kit (QIAGEN, New York, NY, USA) and cloned into the pMD18-T vector (Takara, Dalian, China). Ligations were transformed into *E. coli* DH-5α cells (Takara, Dalian, China) and plasmid DNA isolated from these bacterial clones was sequenced. The confirmed constructs were named p18T-bu*7SK* and p18T-bu*U6*.

The sequences were compared using the Basic Local Alignment Search Tool (BLAST; http://blast.ncbi.nlm.nih.gov/). The putative bu*7SK* and bu*U6* promoters were submitted to GenBank under the accession numbers JN417658 and JN417659.

### 4.2. Construction of *7SK* and *U6* shRNA Expression Vectors

To test the expression competence of the cloned bu*7SK* and bu*U6* promoters, the pMCS-shEGFP and pMCS-sh1864 base vectors were constructed from pMD18T by ligating a 65 bp synthetic copy of an EGFP-specific shRNA or a control scrambled shRNA (Addgene plasmid #1864; Raleigh, NC, USA) sequence produced by chemical synthesis (GenScript, Nanjing, China) between the *Bam*HI and *Hin*dIII sites (Takara, Dalian, China), respectively. Then anti-EGFP shRNA expression cassettes bu*7SK*-shEGFP and bu*U6*-shEGFP were constructed by ligating bu*7SK* and bu*U6* into pMCS-shEGFP, respectively. The negative control expression vector, bu*7SK*-sh1864, was also generated by the same method. To compare the level of expression from pol III promoters of different origin, primers were designed using the reference sequences of h*7SK* (AY578685), h*U6* (AY623053), bo*7SK* (CR810253) and bo*U6* (DQ150531). Then, each promoter was amplified, sequenced and ligated into pMCS-shEGFP using *Xba*I/*Sac*I or *Cla*I/*Xho*I sites introduced in the primers (Table 1). A total of 7 cassettes were constructed: bu*7SK*-shEGFP, bu*U6*-shEGFP, h*7SK*-shEGFP, h*U6*-shEGFP, bo*7SK*-shEGFP, bo*U6*-shEGFP and bu*7SK*-sh1864.

### 4.3. Cell Culture and Transfection

Buffalo fetal fibroblasts (BFF) cells and the mouse PT67 cell line were maintained in Dulbecco Minimal Essential Media (DMEM, GIBCO, New York, NY, USA) with 1% (*w*/*v*) sodium pyruvate and 10% (*v*/*v*) Fetal Calf Serum (FCS, GIBCO, New York, NY, USA). All cells were cultured in a humidified 37 °C atmosphere containing 5% CO_2_. BFF cells and PT67 cells were grown to approximately 80% confluence before transfection. Co-transfection of BFF with 250 ng anti-EGFP shRNA expression plasmid and/or 250 ng pEGFP-N1 in 24-well plates was carried out using Lipofectamine 2000 (Invitrogen, New York, NY, USA) according to the manufacturer’s instructions for fluorescence microscopy observation and flow cytometry analysis. Co-transfection of BFF or PT67 cells with 1.25 μg anti-EGFP shRNA expression plasmid and/or 1.25 μg pEGFP-N1 in 6-well plates was followed by isolation of total RNA for quantification of the expression level of shRNA and EGFP.

The expression of EGFP was observed at 60 h post-transfection. Fluorescence microscopy was performed on duplicate co-transfections using a Nikon Fluorescence Microscope (Nikon, Tokyo, Japan). Images were captured at 40× magnification using a Nikon color digital camera (Nikon, Tokyo, Japan) with the exposure time of 800 msec and compiled with Photoshop CS3 imaging software (Adobe, New York, NY, USA).

### 4.4. Flow Cytometry Assay

To determine the efficiency of EGFP silencing after transfection of the shEGFP constructs, the transfected BFF cells were assayed by flow cytometry. Buffalo fetal fibroblasts (BFF) cells were harvested 72 h post-transfection using 0.25% (*w*/*v*) trypsin-EDTA, pelleted at 1200 rpm for 5 min, washed in phosphate buffered saline (PBS) and resuspended in FACS-solution (1% fetal calf serum (*v*/*v*) in PBS). Analysis was performed using a FACSCalibur (Becton Dickinson, New York, NY, USA) fluorescence-activated cell sorter and CELL Quest software (Becton Dickinson, New York, NY, USA). After flow cytometry, the EGFP Mean Fluorescence Intensity (MFI) of each sample was recorded and compared. The reduction in EGFP MFI for each co-transfection was calculated by normalizing the average MFI (derived from triplicate samples) as a percentage of the MFI of the negative control pbu*7SK*-sh1864/pEGFP-N1 co-transfected cells (100% ± 0.14% (SEM)).

### 4.5. Real-Time PCR Assay

After total RNA was extracted, 2 μg of total RNA was reverse-transcribed to cDNA using AMV reverse transcriptase (Takara, Dalian, China) and Oligo primers (Takara, Dalian, China). Buffalo beta-actin and mouse beta-actin were used as endogenous controls for EGFP expression analysis, while buffalo *U6* was used as the endogenous control for shEGFP expression analysis. Primer pairs were designed to probe shEGFP (5′-ACA CTC CAG CTG GGC TGA CCC TGA AGT TC-3′ and 5′-CTC AAC TGG TGT GTC GTG GAG TC-3′) and EGFP (5′-ACG GCA TCA AGG TGA ACT-3′ and 5′-GTG TTC TGC TGG TAG TGG TC-3′) were employed in this assay. Quantitative real-time PCR (qRT-PCR) was performed using SYBR Green Master (Rox; Roche, Basel, Switzerland) and the Applied Biosystems 7500 Sequence Detection System (Applied Biosystems, New York, NY, USA). Amplification curves were generated with an initial denaturing step at 95 °C for 5 min, followed by 45 cycles of 95 °C for 5 s, 60 °C for 5 s, and 72 °C for 8 s. Melting curves were generated using the following program: PCR products were denatured at 95 °C and cooled to 65 °C at a rate of 20 °C per second. The fluorescence at 530 nm was then recorded continuously from 65 to 95 °C as the temperature was increased at a rate of 0.2 °C per second. All reactions, including the no-template controls, were run in triplicate. After the reactions, the CT values were determined using fixed-threshold settings.

## 5. Conclusions

In summary, we identified and characterized the functional bu*7SK* and bu*U6* promoters suitable for driving the expression of shRNAs in buffalo species. Compared with human, porcine and bovine pol III promoters, the buffalo promoters, directing the expression of EGFP-specific shRNA, showed substantial activity in homologous and heterologous cells. The use of the two promoter sequences and the shRNA vector cloning strategy described here will be advantageous in RNAi functional genomic studies of buffalo cells. Furthermore, the identification and characterization of these promoters is an important step in the development of novel buffalo-specific RNAi-based therapeutics and methods of transgenic delivery of shRNA molecules for future improvements in buffalo properties.

## Figures and Tables

**Table 1.** Primers for cloning of promoters.

Primer name	Sequence
bu*7SK*-F	5′-TCTAGAGAGACAGACCTGGCTCCAC-3′ a
bu*7SK*-R	5′-GAGCTCCACATCCGAGACACTCTGC-3′ b
bu*U6*-F	5′-ATCGATGAGCATTCAGTCCGGCAG-3′ c
bu*U6*-R	5′-CTCGAGGCACGGTAAACATGGCTTC-3′ d
h*7SK*-F	5′-TCTAGACTGCAGTATTTAGCATGCCCC-3′ a
h*7SK*-R	5′-GAGCTCGAGGTACCCAGGCGGC-3′ b
h*U6*-F	5′-ATCGATCCCCCGAGTCCAACAC-3′ c
h*U6*-R	5′-CTCGAGGTGTTTCGTTCTTTCCACAAG-3′ d
bo*7SK*-F	5′-TCTAGAGAGCGTGAGAGACTCGGAGC-3′ a
bo*7SK*-R	5′-GAGCTCCACCATGGTAGTTCGCGCAG-3′ b
bo*U6*-F	5′-ATCGATAAGAGGCTCCTGAGCAACG-3′ c
bo*U6*-R	5′-CTCGAGCATTTACCCCCCACAGAATATATAAG-3′ d

a The underlined sequence indicates *Xba*I restriction endonuclease site;

b The underlined sequence indicates *Sac*I restriction endonuclease site;

c The underlined sequence indicates *Cla*I restriction endonuclease site;

d The underlined sequence indicates *Xho*I restriction endonuclease site.
